# The effect of public reporting presentation on patients’ decision making: An experimental survey in Yunan Province, China

**DOI:** 10.1097/MD.0000000000007203

**Published:** 2017-06-16

**Authors:** Chenxi Liu, Yuqing Tang, Dan Wang, Xinping Zhang

**Affiliations:** School of Medicine and Health Management, Tongji Medical College of Huazhong University of Science and Technology, Wuhan, China.

**Keywords:** comparative performance information, information presentation, logistic regression, public reporting, quality improvement, Western China

## Abstract

Public reporting of comparative performance information (PRCPI) influence patients’ decision making and optimal presentation of PRCPI is urgently required for successful patients’ engagement and quality improvement. This study was to explore the presentation of PRCPI impacting on consumers’ decision making.

This research applied a controlled design, with participants allocated randomly to 6 groups, and a total of 515 participants were recruited in Yunnan province. Five aspects of PRCPI presentations were evaluated, including display (star rating vs numeric information), and whether information was simplified, interpreted, overload, or ranked. Participants were stimulated to identify the best or worst physicians with a hypothetical scenario. Main outcome measures were correct choices rate of best/worst physicians, indicating participants fully understood and correctly used PRCPI. χ^2^ test and logistic regression were applied to assess the effect between different presentations on consumers’ decision making.

The correct choices rate is only 48.93%. Compared with star rating, numerical information helped participants differentiate low-performance physicians (OR = 2.573, *P* = .029), including low-performance physicians in antibiotics (OR = 2.974, *P* = .031) and low-performance physicians in injections (OR = 2.369, *P* = .035). Disordered information impeded participants to fully understand and correctly use PRCPI (OR = 0.519, *P* = .041). The effect was mainly reflected on participants differentiating low-performance physicians (OR = 0.491, *P* = .039) and low-performance physicians in injections (OR = 0.440, *P* = .016). Other aspects of PRCPI showed nonsignificant impacts on consumers’ decision making.

Presentation, including information display and ranking, can influence patients’ correct usage of PRCPI and the effect was mainly observed when the patients were identifying poorly performing physicians. The present study demonstrated that numerical and ranked PRCPI, combined with sufficient patient education, could be most effective to facilitate patient use.

## Abstract 2

Supplemental Digital Content is available in the text

## 1 Introduction

For decades, irrational use of medicines has been considered as a worldwide challenge. It is estimated that over 50% of all medicines are inappropriately prescribed, dispensed, or sold^[1]^ and such practices are more prevalent in low- and middle-income countries (LMICs).^[2]^ In China, overuse of antibiotics and injections is an outstanding inappropriate use of prescribing pharmaceuticals^[3]^, with over 50% of outpatients prescribed,^[4]^ which far exceeds the recommended rate (antibiotics: 30%, injections: 24%). The irrational use of medicines contributes to adverse drug reactions, antimicrobial resistance, protracted illness, and even death.^[5,6]^ It also imposes unexpectedly high financial burden on society^[7]^ and severely hinders quality improvement of health care^[8]^.

Recently, public reporting of comparative performance information (PRCPI) has been used as an important quality-improvement instrument in most developed countries.^[9]^ The USA and UK lead the modern PRCPI movement,^[10,11]^ which is proposed to spark consumers to make high-quality health care choices.^[12]^ However, little evidence has been reported, to date, that PRCPI influences patients’ selection of providers.^[13]^

The complexity of the reports’ content and design plays an important role in failure of public reporting.^[14–16]^ The most frequently discussed barrier is that consumers do not understand the formats in which the information is presented.^[17]^ The 2011 National Summit on public reporting, sponsored by the Agency for Health care Research and Quality (AHRQ), concluded that changes in the presentation of future PRCPI is vital and urgent, and the optimal content, structure, and communication vehicle of PRCPI for successful patients’ engagement is still veiled.^[18]^ To bridge the gap between PRCPI presentation and consumers’ usage, there is increasing research focusing on design of PRCPI that optimize consumers’ use of performance information these years^[12]^ and the essential requirement for effective PRCPI is to present the information less complicatedly. However, the impact of PRCPI presentation was not concluded.

Although, a few of researches have been conducted in the United States and other developed countries,^[12]^ there is scarce evidence concerning effective PRCPI presentation in LMICs, in which patients are poorly educated and of poor socioeconomic status. As an inevitable trend worldwide, many LMICs have applied PRCPI to improve the quality of care and it is essential to understand the effect of different PRCPI presentations to facilitate effective PRCPI policy.^[19]^ Specifically, the objective of the present research is to explore how PRCPI presentation impacts consumers’ decision making and to add the evidence for optimal PRCPI presentation in LMICs. Ultimately, this study provides an empirical basis for enhancing public reporting effect.

## 2 Methods

### 2.1 Setting

This study was conducted in Lijiang City, Yunnan Province, which is located in West China. Because of underdeveloped economy and poor health literacy of residents, the quality of care in West China is relatively low and urgently needs to be improved. Yunnan province has 45 million residents and ranks in the middle range among all Chinese provinces in terms of population. Lijiang is situated in the northwest portion of Yunnan Province. The government of Yunnan Province has been highly supportive of PRCPI. To provide evidence for PRCPI policy in West China, Lijiang was selected as our research site.

The study was approved by the Ethical Review Committee of Tongji Medical College, Huazhong University of Science and Technology (No. IORG0003571) before the start of the study, and written informed consent was obtained. Each participant was given an umbrella (nearly $3.08) for participation.

### 2.2 Hypothesis and group design

Based on the previous research,^[12]^ several characters played an important role in facilitating consumers’ choice, including number of choices,^[20–24]^ information simplifying,^[25,26]^ order,^[27,28]^ display type,^[27,29–31]^ etc. Thus, several presentations of PRCPI were extracted and the following hypotheses were intended to test:

- H1: the effect of star rating on patients’ decision making;
- H2: the effect of numerical information on patients’ decision making;
- H3: the effect of simplified information on patients’ decision making;
- H4: the effect of insufficient knowledge on patients’ decision making;
- H5: the effect of overloaded information on patients’ decision making;
- H6: the effect of ranking on patients’ decision making.

Six groups, matching the hypotheses respectively, were developed to explore the effect among different presentations. The design of group referred to principles of randomization, control, and blinding. Participants will be randomly divided into 6 groups based on the sequence they enter into the research and all participants were unaware of the group to which they will be assigned. The details of group designs are shown in Table 1.

**Table 1:**
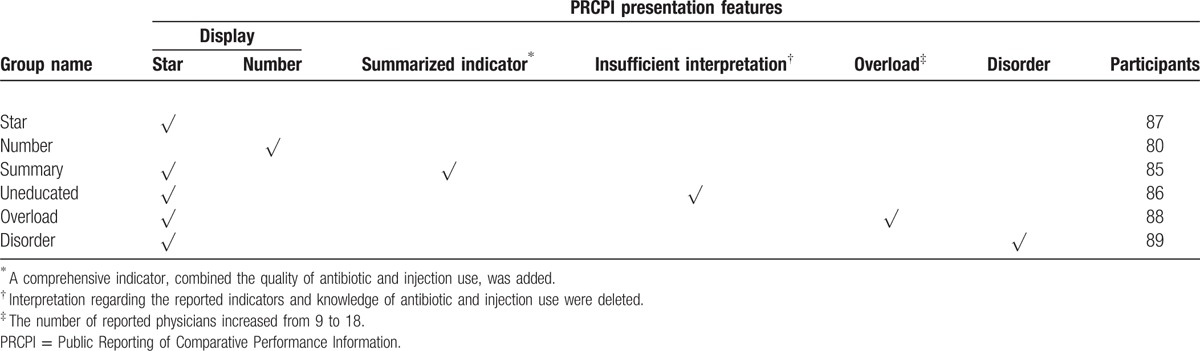
Design of different kinds of PRCPI presentation.

As star rating is a common visual display of provider performance information in different public reporting systems,^[27]^ such as Nursing Home Compare and Home Health Compare, this kind of display was applied in all groups except for the number group.

Because overuse of antibiotics and injections is severely prevalent in West China, antibiotic prescription rates and injection prescription rates were selected as content of released information. (The specific PRCPI materials of different groups could be seen in Supplemental File 1).

### 2.3 Sample

Based on the previous experimental design,^[29,32,33]^ a total of 480 individuals, 80 for each group, were determined in the study. To ensure the sufficient sample size, 540 participants were recruited. Moreover, to ensure participants had the potential to use the releasing information, the basic literacy was required. The inclusion criteria were: participants have completed junior middle school; participants have the basic literacy to read the materials. participants have no ophthalmic diseases that they have troubles to see the information. Participants were recruited from 3 districts which were randomly selected, including Minzhu, Shuhe, and Xi’an districts.

### 2.4 Scenario design

In this study, a hypothetical scenario was presented and all participants were asked to choose the best physicians for themselves. More specifically, participants read the following paragraphs and were asked to choose a physician for treatment based on the information presented to them:

Imagine that you have caught a mild cold for 2 days and want to visit a primary care institution to see a physician. There are several physicians available to choose in primary care institutions and you are not sure how and which to choose among the different physicians. Upon arrival, a 1.2 m × 0.8 m poster is displayed on a bulletin board in the lobby of primary care. In the poster, you will see information about prescribing information for each physician (name, estimated antibiotic prescription rate, estimated injection prescription rate, etc). Please try to make the best choice and identify which physician should avoid visiting.

After reading a scenario description, participants were instructed to answer the following questions one by one. Once they completed a question, the answer could not be revised.

Q1. Choose the best physician for antibiotic prescribing?

Q2. Choose the worst physician for antibiotic prescribing?

Q3. Choose the best physician for injection prescribing?

Q4. Choose the worst physician for injection prescribing?

Q5. Choose the best physician whom you want to see.

Q6. Choose the worst physician whom you should avoid to see.

Before determining the final design of the scenario and questions, we conducted a pilot survey in Qian Jiang City, Hubei Province. The statements were modified to present unambiguous questions and scenario based on the feedback from pilot survey. (The specific scenario description and questionnaire could be seen in Supplemental file 2).

### 2.5 Data collection

This study was conducted from March 10 to 15, 2015. It required an average of 20 minutes to complete the reading and following questions. Participants were randomly divided into 6 groups based on the sequence they entered into the research. All participants were unaware of the group to which they were assigned. A total of 515 participants had completed the survey (seeing in Table 1). Participant demographic information, such as gender, age, literacy, health condition, and family income per year were also collected.

### 2.6 Data analysis

A total of 7 indicators, correct choices rate of best/worst physicians, were treated as outcome measurements. Six indicators measured whether each participant selected the correct answer to Q1–Q6 and a synthesized indicator measured whether participant fully understood and correctly used PRCPI.

For Q1, Q3, and Q5, the criteria for the correct choice was that the best physician marked with 3 stars ((★★★)) or lowest injection/antibiotic prescription rate was selected (showed by number); for Q2, Q4, and Q6, the worst physician was marked with 1 star ((★)) or highest injection/antibiotic prescription rate (showed by number). For the synthesized indicator, participant’ choices which were all right to Q1–Q6 was considered as full understanding and usage of PRCPI.

Frequencies were generated for correct and incorrect answers. The comparison between the groups was calculated by χ^2^ test/Fisher exact test and star group was chosen as the baseline.

To quantify the effect of different presentations, a logistic regression analysis was conducted for each outcome measurement. Gender, age, and literacy were included as adjustment to control the confounding effect.^[32]^ Each participant was considered an analytic unit. Where group is a dummy variable and β1 represented the effect of different presentations. Group 1 was chosen as the baseline. For each patient, the probability *P* of the occurrence of a dichotomous outcome *Y* can be described as

Logistic (*P*) = β0 +β1 × Group +β2 × Gender +β3 × age +β4 × Literacy + ε

All statistical analyses were conducted with STATA version 10.0. Statistical significance was set at *P* <.05 and all *P*-values reported were 2-tailed.

## 3 Results

### 3.1 Demographic information of the participants

A total of 515 participants consented to participate in the study and there were at least 80 participants in each group (see Table 1). The mean age of all participants were 29 years (SD: ±9.19), and over half of them were females (52.82%). The literacy of most participants corresponded to vocational school (30.29%) or senior high school level (26.41%). Among all the participants, 78.45% believed that their health condition were either excellent or good. The annual household income of 55.15% participants was below ¥100,000 (approximately $15,730). The detail of demographic information is shown in Table 2.

**Table 2:**
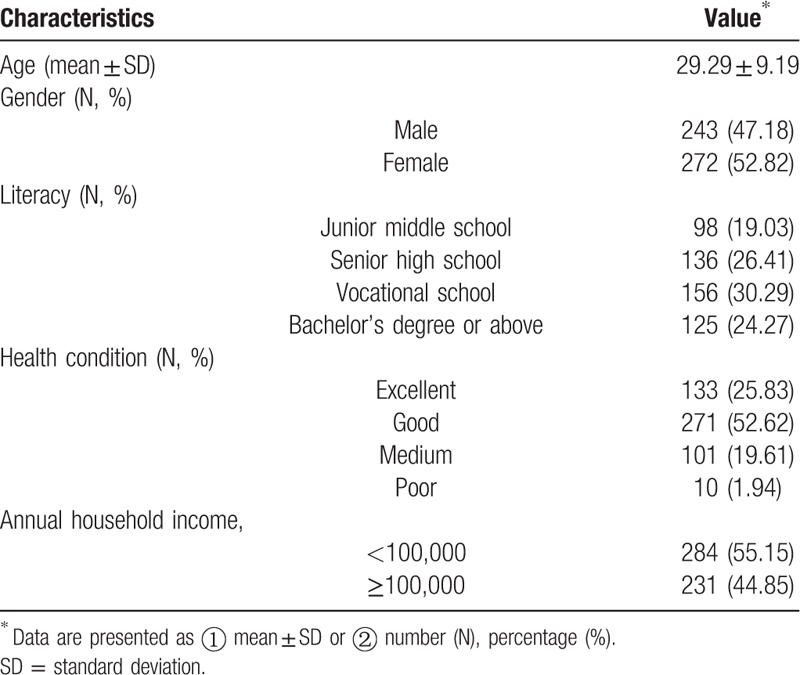
Demographic information of the participants.

### 3.2 Correct choices rate of PRCPI

Table 3 presented the results of correct answer rate of participants to each question in each group. As a whole, high rate of correct usage of PRCPI was noted, with around 70% correct answer to each single question (Q1–Q6). However, summarized indicator showed that only 48.93% participants fully understood and correctly used PRCPI.

**Table 3:**
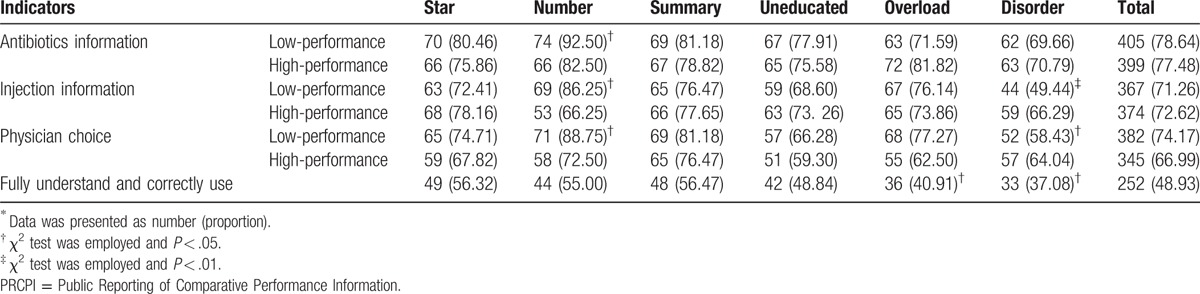
correct choices proportion of PRCPI in each group^∗^.

The χ^2^ test was employed to evaluate the difference between groups. Compared with star rating, numerical information helped participants distinguish physicians with poor performance more effectively. The differences were statistically significant in antibiotics choice (*χ*^2^: 5.0875, *P* = .024), injections choice (*χ*^2^: 4.8163, *P* = .028), and physicians choice (*χ*^2^: 5.4325, *P* = .020). However, the proportion of participants who fully understood and correctly used all PRCPI was not significantly different.

In the overload group, though there was no difference for each single question (Q1–Q6), only 40.91% participants could fully understand and correctly use PRCPI and the result was statistically significant (*χ*^2^: 4.1604, *P* = .041) compared with the star group.

Besides, disorder information hindered participants respond correctly compared with the star group. Only 49.44% of participants distinguished poor-performance physicians in injection prescribing (*χ*^2^: 9.7437, *P* = .002), and only 58.43% of participants distinguished worst-performance physicians (*χ*^2^: 5.2360, *P* = .022). Participants in other groups (summary and uneducated) showed nonsignificant differences. The details of the results are showed in Table 3.

### 3.3 Effect of different presentations on participants’ correct usage of PRCPI

Logistic regression model was applied to quantify the effects of different presentations. Participants’ demographic characters, including age, gender, and literacy, were included for adjustment. The considering group was a dummy variable, star group was treated as the controlled group.

There was no statistical difference when participants chose high-performance physicians in all groups. However, the effect of different presentation was found when participants distinguished low-performance physicians.

Compared with presentation of star rating, numerical information helped participants use PRCPI more accurately to differentiate low-performance physicians. After adjustment for demographic variables, the results showed that numerical information significantly increased participants’ ability to distinguish low-performance physicians (OR = 2.573, *P* = .029). Similar effect was also reported in distinguishing low-performance physicians in antibiotics (OR = 2.974, *P* = .031) and low-performance physicians in injections (OR = 2.369, *P* = .035).

Disordered information impeded participants to fully understand and correctly use PRCPI (OR = 0.519, *P* = .041). The effect was mainly reflected on participants differentiating low-performance physicians (OR = 0.491, *P* = .039) and low-performance physicians in injections (OR = 0.440, *P* = .016).

After controlling demographic characters, overload information was no longer hindering participants to fully understand and correctly use PRCPI (OR = 0.597, *P* = .107). Other aspects of PRCPI showed nonsignificant impacts on consumers’ decision making. The details of the regression analysis are shown in Table 4.

**Table 4:**
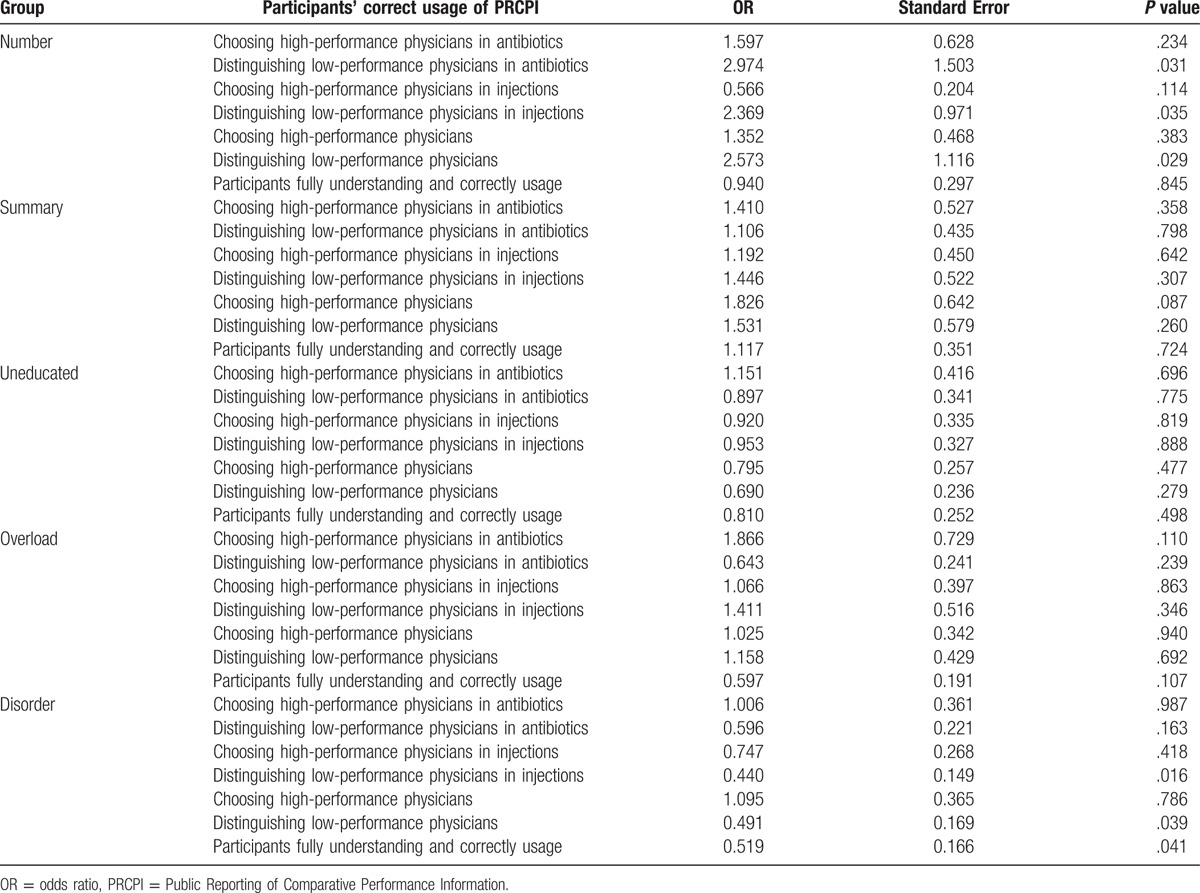
Effect of different presentations on participants’ correct usage of PRCPI.

## 4 Discussion

This study demonstrated that PRCPI presentation can assist participants in choosing healthcare providers on a formal test in Western China. The influencing features were display and whether the information was ordered. Furthermore, the effect was mainly observed when patients were identifying poorly performing physicians.

### 4.1 The effect of different displays of PRCPI

Star rating is a common visual display of provider performance information in different countries.^[27]^ Compared with star ratings, numerical information helped participants differentiate poorly performing physicians. The results were contradiction with the previous research in the psychology field, in which presenting numerical data often included difficult types of information to understand.^[34,35]^ However, in medical information dissemination, a similar effect was reported when public reported drug adverse events (DAEs). Individuals using non-numeric formats information over- or underestimated DAEs risks substantially more than those provided numeric formats.^[31]^ It seemed that non-numeric presentation may mislead the patients’ assessment and interpretation of information. In the present research, star was considered as positive evaluation in general and would confuse participants when they had to indicate poorly performing physicians.

### 4.2 The effect of ranking of PRCPI

Notably, disordered performance information impeded participants to fully understand and correctly use PRCPI. The results confirmed the previous findings in the USA, in which more effective use was found when health plans were ranked by performance.^[36,37]^ However, research from the Netherland reported that an alphabetical ordering of providers facilitated consumers’ effective use compared with ordering of providers’ performance information. The possible explanation is that American citizens are more accustomed to rankings of performance while Netherlanders are more used to alphabetical ordering.^[27]^ Overall, ordering no matter by performance or alphabet, providing visual cues, was an effective vehicle to facilitate patients’ comprehension and use of information.^[36,37]^

### 4.3 The effect to identifying physicians with poor performance

In our research, different presentations, aiming at simplifying released information, could not influence patients’ identification of well-performing physicians and the effect was only found when participants had to indicate the worse providers. The possible reason for the results was the participants’ interest for different questions. In the area of pedagogy, Klare and George^[38]^ had shown that different levels of motivation could skew the results of text comprehension. When readers were poorly motivated, comprehension could be improved by simplification of information, while the effect was limited when readers were of high motivation.^[39]^ Compared with differentiating well-performing physicians, participants have less interest to identify the poorer one. Previous evidence found similar results that a combination of bar chart and star ratings helped consumers identify low-quality providers, while the choice of best provider was not affected by any of the presentation features.^[27]^ Presentation of PRCPI was more important when consumers’ interest was low than when it was high.

### 4.4 The potential mechanism for different presentation of PRCPI

Most existing research works, aiming at exploring optimal PRCPI, were based on the mechanism that improved presentation of PRCPI that could boost patients’ comprehension and selection and ultimately change market share of providers.^[16,40]^ They examined the effect of presentation using consumers’ correct choice rate on well-performing providers and found no significant differences.^[36,37]^ According to existing results, the mechanism for presentation influencing PRCPI should focus on increased patient capability to differentiate poorly performing providers. Furthermore, PRCPI also imposes a threat in providers’ reputation and help them identify and improve areas in which they underperform.^[10,16,40]^ Improved PRCPI presentations would put heavier pressure on providers and the relationship between PRCPI presentations and providers’ changes may need further research.

### 4.5 The effect of overload of PRCPI

In the present research, participants’ selection was not affected by overloaded choices’ size of physicians. One study, for example, found one-third lower probability of choosing the high-quality health plan when participants went from 3 to 9 choices.^[41]^ Previous studies has showed that optimal number of choices that follows an inverted “U” shape—as the number increases, respondents’ decision quality initially increases and then decreases.^[23,42]^ In our study, the choice size doubled (from 9 to 18) and we believed that it was overwhelming for participants with limited health literacy. The possible reason was that ordering by performance would reduce information processing load for participants and hinder the effect of overloaded choices.

As the Chinese governments have educated the public about risk of antibiotic and injection overuse recently, at least people have the basic knowledge toward rational use of medicine. The summarized indicator and basic knowledge of PRCPI, aiming at reducing misinterpretation, showed limited effect on participants’ choices. However, according to the previous experimental research in the United States, participants’ correct comprehension rate to PRCPI was 77% and the effective use of PRCPI was 88.4%.^[27]^ These results were much higher compared with Western China residents, with 73.52% and 48.93%, respectively. Patient education and optimal presentation were still imperative for Western China citizens. Suggestion for presenting information about rational use of medicine in Western China is numerical information, combining with ordering and sufficient patient education.

In the present study, the scenario was simulated, information was provided, and participants were healthy people with basic literacy. It is quite different compared with the situation in the real world in which patients always have to seek health information themselves.^[43]^ Individual disability, such as physical immobility, ophthalmic diseases, and hearing impairment, put extra challenges for patient access to PRCPI. Even when information was obtained, lack of basic literacy skills will pose a heavy burden to patients’ understanding and using of PRCPI.^[44]^ In addition, whether patients would eventually use PRCPI to choose health providers rely on many other factors, including the perceived value of information, characteristics of patients (belief, race, and culture), and external factors such as the health care system arrangements.^[44]^ When health policy was designed, using PRCPI as an intervention to improve quality of care, all these aspects should be carefully considered.

## 5 Limitation

First, the experimental study required basic literacy. All participants had completed junior middle school and the effect of the PRCPI presentation may be overestimated when compared with the real-world population. Second, the study sites were in Western China, therefore, the conclusions drawn from this research must be carefully generalized to other regions.

## 6 Conclusion

Presentation, including information display and ranking, can influence patients’ correct usage of PRCPI and the effect was mainly observed when the patients were identifying poorly performing physicians. The study confirmed that PRCPI should be carefully designed to spark patient to make informed healthcare decision. Considering that PRCPI is increasingly used as a common instrument for quality improvement worldwide, optimal presentation is still veiled. The present study demonstrated that numerical and ranked PRCPI, combined with sufficient patient education, could be most effective to facilitate patient use. To help address irrational use of medicines, PRCPI has the potential to change the prevalent misunderstanding among patients toward antibiotics and injections in China as well as other LMICs by providing knowledge and more transparency in a way that patients can easily understand and use. PRCPI also imposes a threat to providers’ reputation and help them identify and improve areas in which they underperform. Through the mechanism, PRCPI, with effective presentation, is promising to achieve the goal of quality improvement.

## Acknowledgments

The authors would like to sincerely thank participants and are also grateful for the support from the local governments.

## Supplementary Material

Supplemental Digital Content
