# Supplementary material for: The effect of public reporting presentation on patients’ decision making: An experimental survey in Yunan Province, China
Source: Medicine (Baltimore). 2017 Jun 16;96(24):e7203. doi: 10.1097/MD.0000000000007203 (PMC5478351; doi:10.1097/MD.0000000000007203)
Supplement: Supplemental Digital Content [file medi-96-e7203-s001.doc]

Supplemental File 1—Public Reporting of Comparative Performance Information for Different Groups

**Publicly released comparative performance information**

(Star Group)

| **Physicians** | **Antibiotic prescribing** | **Injection prescribing** |
| --- | --- | --- |
| **A** | ★★★ | ★★★ |
| **B** | ★★★ | ★★★ |
| **C** | ★★★ | ★★ |
| **D** | ★★ | ★★★ |
| **E** | ★★ | ★★ |
| **F** | ★★ | ★★ |
| **G** | ★ | ★★ |
| **H** | ★ | ★★ |
| **I** | ★ | ★ |

**Indicator interpretation:**

Three stars (★★★) was considered as low frequency of injections/antibiotics use.

Two stars (★★) was considered as middle frequency of injections/antibiotics use.

One star (★) was considered as high frequency of injections/antibiotics use.

For example, compared with three star physicians, two star physicians are more likely to prescribe antibiotics/injections for you.

**Basic knowledge of rational use of medicines:**

In China, it is estimated that over 50% patients were prescribed with antibiotics and injections. The recommended percentage is 13.4%– 24.1% for rate of antibiotic use and 20.0%– 26.8% for rate of injection use in primary care (World Health Organization). In primary cares, over 80% patients have antibiotics prescription when they don’t need to and this value is more than 70% for injections prescriptions.

**Publicly released comparative performance information**

(Number Group)

| **Physicians** | **Antibiotic prescribing rate** | **Injection prescribing rate** |
| --- | --- | --- |
| **A** | **30%** | **30%** |
| **B** | **30%** | **40%** |
| **C** | **30%** | **50%** |
| **D** | **50%** | **40%** |
| **E** | **50%** | **50%** |
| **F** | **50%** | **60%** |
| **G** | **70%** | **50%** |
| **H** | **70%** | **60%** |
| **I** | **70%** | **70%** |

**Indicator interpretation:**

**Antibiotic prescribing rate** = Number of antibiotic prescriptions / total number of a physician’s prescriptions × 100%; **Injection prescribing rate** = Number of injection prescriptions / total number of a physician’s prescriptions ×100%; For example, if the antibiotic prescribing rate is 60% for physician E, it means that 60% of physician E’s patients have been prescribed with antibiotics before.

**Basic knowledge of rational use of medicines:**

In China, it is estimated that over 50% patients were prescribed with antibiotics and injections. The recommended percentage is 13.4%– 24.1% for rate of antibiotic use and 20.0%– 26.8% for rate of injection use in primary care (World Health Organization). In primary cares, over 80% patients have antibiotics prescription when they don’t need to and this value is more than 70% for injections prescriptions.

**Publicly released comparative performance information**

(Summary Group)

| **Physicians** | **Antibiotic prescribing** | **Injection prescribing** | **Comprehensive prescribing** |
| --- | --- | --- | --- |
| **A** | **★★★** | **★★★** | **★★★** |
| **B** | **★★★** | **★★★** | **★★★** |
| **C** | **★★★** | **★★** | **★★** |
| **D** | **★★** | **★★★** | **★★** |
| **E** | **★★** | **★★** | **★★** |
| **F** | **★★** | **★★** | **★★** |
| **G** | **★** | **★★** | **★** |
| **H** | **★** | **★★** | **★** |
| **I** | **★** | **★** | **★** |

**Indicator interpretation:**

Three stars (★★★) was considered as low frequency for injections/antibiotics use and high quality for comprehensive prescribing.

Two stars (★★) was considered as middle frequency of injections/antibiotics use and middle quality for comprehensive prescribing.

One star (★) was considered as high frequency of injections/antibiotics use and poor quality for comprehensive prescribing.

For example, compared with three star physicians, two star physicians are more likely to prescribe antibiotics/injections for you.

**Basic knowledge of rational use of medicines:**

In China, it is estimated that over 50% patients were prescribed with antibiotics and injections. The recommended percentage is 13.4%– 24.1% for rate of antibiotic use and 20.0%– 26.8% for rate of injection use in primary care (World Health Organization). In primary cares, over 80% patients have antibiotics prescription when they don’t need to and this value is more than 70% for injections prescriptions.

**Publicly released comparative performance information**

(Uneducated Group)

| **Physicians** | **Antibiotic prescribing** | **Injection prescribing** |
| --- | --- | --- |
| **A** | ★★★ | ★★★ |
| **B** | ★★★ | ★★★ |
| **C** | ★★★ | ★★ |
| **D** | ★★ | ★★★ |
| **E** | ★★ | ★★ |
| **F** | ★★ | ★★ |
| **G** | ★ | ★★ |
| **H** | ★ | ★★ |
| **I** | ★ | ★ |

**Indicator interpretation:**

Three stars (★★★) was considered as low frequency of injections/antibiotics use.

Two stars (★★) was considered as middle frequency of injections/antibiotics use.

One star (★) was considered as high frequency of injections/antibiotics use.

For example, compared with three star physicians, two star physicians are more likely to prescribe antibiotics/injections for you.

**Publicly released comparative performance information**

(Overload Group)

| **Physicians** | **Antibiotic prescribing** | **Injection prescribing** |
| --- | --- | --- |
| **A** | **★★★** | **★★★** |
| **B** | **★★★** | **★★★** |
| **C** | **★★★** | **★★★** |
| **D** | **★★★** | **★★★** |
| **E** | **★★★** | **★★** |
| **F** | **★★★** | **★★** |
| **G** | **★★** | **★★★** |
| **H** | **★★** | **★★★** |
| **I** | **★★** | **★★** |
| **J** | **★★** | **★★** |
| **K** | **★★** | **★★** |
| **L** | **★★** | **★★** |
| **M** | **★** | **★★** |
| **N** | **★** | **★★** |
| **O** | **★** | **★★** |
| **P** | **★** | **★★** |
| **Q** | **★** | **★** |
| **R** | **★** | **★** |

**Indicator interpretation:**

Three stars (★★★) was considered as low frequency of injections/antibiotics use.

Two stars (★★) was considered as middle frequency of injections/antibiotics use.

One star (★) was considered as high frequency of injections/antibiotics use.

For example, compared with three star physicians, two star physicians are more likely to prescribe antibiotics/injections for you.

**Basic knowledge of rational use of medicines:**

In China, it is estimated that over 50% patients were prescribed with antibiotics and injections. The recommended percentage is 13.4%– 24.1% for rate of antibiotic use and 20.0%– 26.8% for rate of injection use in primary care (World Health Organization). In primary cares, over 80% patients have antibiotics prescription when they don’t need to and this value is more than 70% for injections prescriptions.

**Publicly released comparative performance information**

(Disorder Group)

| **Physicians** | **Antibiotic prescribing** | **Injection prescribing** |
| --- | --- | --- |
| **A** | **★★** | **★★** |
| **B** | **★** | **★** |
| **C** | **★★★** | **★★** |
| **D** | **★** | **★★** |
| **E** | **★★** | **★★★** |
| **F** | **★★** | **★★** |
| **G** | **★★★** | **★★★** |
| **H** | **★★★** | **★★★** |
| **I** | **★** | **★★** |

**Indicator interpretation:**

Three stars (★★★) was considered as low frequency of injections/antibiotics use.

Two stars (★★) was considered as middle frequency of injections/antibiotics use.

One star (★) was considered as high frequency of injections/antibiotics use.

For example, compared with three star physicians, two star physicians are more likely to prescribe antibiotics/injections for you.

**Basic knowledge of rational use of medicines:**

In China, it is estimated that over 50% patients were prescribed with antibiotics and injections. The recommended percentage is 13.4%– 24.1% for rate of antibiotic use and 20.0%– 26.8% for rate of injection use in primary care (World Health Organization). In primary cares, over 80% patients have antibiotics prescription when they don’t need to and this value is more than 70% for injections prescriptions.

Supplemental File 2—Scenario Description and Survey Questionnaire

Scenario description for survey

| Dear Ms. / Mr.:  Good day!  Thank you very much for your participation in our investigation, and we will keep your basic information confidential strictly.  Here is a simulated scenario as following and all survey will last for around 20 minutes:  *Image that you have caught a mild cold for two days and want to visit a primary care institution to see a physician. There are several physicians available to choose in primary care institutions and you are not sure how and which to choose among the different physicians. Upon arrival, a 1.2m × 0.8m poster is displayed on a bulletin board in the lobby of primary care. In the poster, you will see information about prescribing information for each physician (name, estimated antibiotic prescription rate, estimated injection prescription rate, and etc). Please try to make the best choice and identify which physician should avoid visiting.* |
| --- |

**Survey questionnaire**

Based on the information showed above, please answer the following questions.

Q1. Choose the **best** physician for **antibiotic prescribing**?

_______________________

Q2. Choose the **worst** physician for **antibiotic prescribing**?

_______________________

Q3. Choose the **best** physician for **injection prescribing**?

_______________________

Q4. Choose the **worst** physician for **injection prescribing**?

_______________________

Q5. Choose the **best** physician **whom you want to see**.

_______________________

Q6. Choose the **worst** physician **whom you should avoid to see**.

_______________________

Finally, please fill in your demographic information

**1. Gender:** A. Male; B. Female.

**2. Age:** ________

**3. Literacy:** A. Junior middle school; B. Senior high school; C. Junior college education; D. Bachelor degree or above

**4. Health condition:** A. Excellent; B. Good; C. Medium; D. Poor; E. Very poor.

**5. Household income per year:** ________
